# Aging in the Skeletal Muscle Revealed by Molecular Immunohistochemical Imaging

**DOI:** 10.3390/ijms26135986

**Published:** 2025-06-22

**Authors:** Manuela Malatesta, Barbara Cisterna

**Affiliations:** Department of Neurosciences, Biomedicine and Movement Sciences, University of Verona, 37134 Verona, Italy; barbara.cisterna@univr.it

**Keywords:** sarcopenia, myonuclei, sarcoplasmic reticulum, sarcolemma, mitochondria, satellite cells, neuromuscular junction, extracellular matrix, vascularization, immunolabelling

## Abstract

The skeletal muscle is a complex organ mainly composed of multinucleated fibres responsible for contractile activity, but it also contains postnatal myogenic stem cells (i.e., satellite cells), connective cells and nervous cells. The skeletal muscle is severely affected by aging, undergoing a progressive reduction in muscle mass, strength and endurance in a condition known as sarcopenia. The mechanisms underlying sarcopenia still need to be completely clarified, but they are undoubtedly multifactorial, involving all cell types constituting the skeletal muscle. Immunohistochemistry has widely been used to investigate skeletal muscle aging, identifying age-related molecular alterations in the various myofibre components, as well as in the satellite cells and peri-fibre environment. The wide range of immunohistochemical data reported in this review is proof of the primary role played by this long-established, yet modern, technique. Its high specificity for the molecules of interest, and the possibility of imaging and quantifying the signal in the real histological or cytological sites where these molecules are located and active, makes immunohistochemistry a unique and irreplaceable tool among the laboratory techniques in biomedicine.

## 1. Introduction

The skeletal muscle is a complex organ in which multinucleated fibres are the main component and active elements related to movement. The muscle fibres comprise several fibre types differing in their functional and metabolic properties [1,2,3]. Besides the multinucleated fibres, the skeletal muscles also contain mononucleated postnatal myogenic stem cells (the so-called satellite cells) and other mononucleated non-muscular cell populations such as connective cells (e.g., fibroblasts, vascular endothelial cells, immune cells) and neuronal and glial Schwann cells, as well as mesenchymal non-myogenic stem cells (e.g., fibro-adipogenic progenitors) [4,5].

The skeletal muscle is severely affected by aging, which involves a progressive reduction in the muscle mass paralleled by a decrease in its strength and endurance. Even physically active healthy subjects suffer this phenomenon, which in humans starts at the age of 30–50 and causes 1% to 8% muscle loss per year [6,7,8]. This state is also known as sarcopenia [9,10] and involves several negative consequences, such as a reduced functional performance with an increased risk of falls and impaired motor, metabolic and physiological functions [10,11,12,13,14]. Thus, sarcopenia in aged subjects is a significant risk factor for weakness, physical disability, loss of independence and premature death.

The mechanisms underlying age-related skeletal muscle wasting and weakness still need to be completely clarified, but they are undoubtedly multifactorial and likely involve all cell types constituting the skeletal muscle. Several hypotheses have been formulated, pointing to muscle tissue factors such as dysfunctional mitochondria [15,16], impaired calcium homeostasis [17,18,19], decreased myofibre nuclei [20,21,22] and quantitative and/or functional alteration of satellite cells [22,23,24,25,26], as well as to other factors such as the degeneration of neuromuscular junctions (NMJ) and myofibre denervation [26,27,28,29,30], reduced microvasculature [31,32] and metabolic and anabolic dysfunctions [33,34,35].

In recent decades, sarcopenia has become a hot topic in scientific research due to the need to set up efficient therapeutic approaches and face the serious healthcare and social problems related to the rising aging population. Among the various methodological approaches used by researchers in their studies on sarcopenia, immunohistochemistry has proved to be an especially versatile and informative technique.

In immunohistochemistry, labelled antibodies are used to detect antigens in the very place where they are present in cells and tissues. The sites where the antibody binds to the antigen are made visible under light or electron microscopy by a suitable marker.

Based on the pioneering works on antibody–antigen recognition by renowned scientists such as Emil von Behring, Paul Erlich, John R. Marrack and Michael Heidelberger (reviewed in Ref. [36]), Albert H. Coons was the first to label anti-pneumococcal antibodies with the fluorescent reagent fluorescein isocyanate, thus being able to detect bacteria ingested by macrophages [37]. This seminal paper actually marked the beginning of immunofluorescence, which, for a couple of decades, became the method of choice to recognize the presence of specific proteins. The development, in the mid-1960s, of histochemical techniques for the detection of enzyme activity led histochemists such as Graham and Karnovsky [38] to successfully attach an enzyme to the antibody, which was made visible after the reaction with the enzyme-specific substrate; as a result, coloured or electron-dense reaction products were obtained at the sites where the reaction did occur [39]. Subsequently, numerous fluorophores have been developed to label antibodies and make them visible under fluorescence microscopy, while colloidal gold has become the marker of choice for immunolabelling in transmission electron microscopy.

There is no doubt that, over the last sixty years, immunohistochemistry has become the most widely used histochemical technique in the biomedical field, with countless applications for basic and applied research in cell biology and especially human pathology [40]. Compared to other biochemical or molecular tests, immunohistochemistry has a unique and distinctive feature as it is performed preserving the original histological architecture: this allows scientists to detect the expression pattern of single molecules (generally proteins) in their microenvironment. Nowadays, immunohistochemistry is used to perform molecular analyses in situ using different imaging techniques and to detect cell phenotype differences that may occur even in limited portions of a tissue or in a few cells of a heterogeneous population.

In the present review article, we aim at highlighting the crucial role of immunohistochemistry in elucidating the cellular and molecular mechanisms involved in skeletal muscle aging. To do this, we focus on investigations performed on the whole skeletal muscle in order to demonstrate how the histochemical use of labelled antibodies may have a dual relevance, their being useful histological markers (to recognize specific cellular and extracellular molecules in a complex organ) as well as unique molecular indicators of cell functional activity in situ. An original comprehensive overview on age-related modifications of both the muscular and non-muscular histological components of the skeletal muscle is provided, reporting detailed information on the various myofibre organelles (sarcolemma, sarcoplasmic reticulum, mitochondria, myonuclei), the satellite cells, and the peri-myofibre structures (NMJ, vasculature and extracellular matrix, ECM).

## 2. Myofibres

### 2.1. Sarcomere

In skeletal muscle fibre, the sarcomere represents the contractile unit, which is limited by two Z-lines [41]. The sarcomeres are arranged in series and form the contractile myofibril [42]. The Z-line is the anchoring site of the thin filaments, composed of actin, tropomyosin and troponin, as well as of the actin crosslinker α-actinin, the principal thick filament supporter titin and several intermediate filaments including desmin and vimentin [43]. The thin filaments are interdigitated with the thick filaments, which are composed mainly of myosin with the addition of the elastic protein titin and the myosin binding protein C [43]. According to the sliding filament theory of muscle contraction, the extent of the generated contractile force depends on the overlapping of the thick and thin filaments and, hence, on the number of the formed cross-bridge interactions.

Evidence exists that the thick filament lengths are constant, whereas the length of the thin filaments may vary, with resulting variations in sarcomere length–tension (reviewed in Ref. [44]). Gokhin et al. [45] used an immunohistochemical approach to investigate in young (2-month-old) and aged (24-month-old) mice the lengths of thin filaments in muscles where the myofibre types and architecture are different, i.e., the tibialis anterior, extensor digitorum longus and soleus muscles. The authors demonstrated that no age-related difference in the thin filament length nor myofibril disorganization and/or deterioration occurred in the tibialis anterior and extensor digitorum longus muscles. On the contrary, a decrease in the thin filament length was observed in the soleus muscle, thus indicating that a muscle-specific age-associated modulation of the thin filament length may take place.

The thick filaments are mainly made of myosin II, which is the specific skeletal muscle myosin isoform, consisting of a globular head and a tail [41]. Myosin II is an adenosine triphosphate (ATP)-dependent protein and, in the “swinging lever-arm” hypothesis of muscle contraction, the movements of the myosin ATPase catalytic site along with rotation of the myosin “neck” promote the sarcomere shortening [46], thus generating movement and force along the thin filaments. Skeletal muscle myosin has a hexameric structure consisting of two myosin heavy chains (MyHCs) and various myosin light chains (MLCs). In several mammalian species (e.g., mice and rats), four main types of fibres, called type 1, 2A, 2X and 2B, are distinguished, based on the presence of specific isoforms of the MyHC [47] and on their oxidative/glycolytic metabolism (type 1 and 2A fibres being more oxidative and type 2B fibres more glycolytic). On the other hand, in most human muscles, only type 1, 2A and 2X fibres are present [48].

Myosin ATPase histochemistry has long been the technique of choice for typing fast, slow and mixed fibres [49], but in more recent years the immunolabelling of the heavy chain of the fast or slow myosin isoforms has at least partially replaced this method [50]. By using both techniques, Kirkeby and Garbarsch [51] histomorphometrically compared the age-related changes in the vastus lateralis and masseter muscles of young (18–24 years) and very old (90–102 years) humans. Although the fibre-type distribution patterns obtained by the two methods did not perfectly overlap, the authors demonstrated that the two muscles are differently affected by aging: in fact, muscular atrophy (resulting in decreased type II fibre and more angulated fibres) occurred to a greater extent in the vastus lateralis than in the masseter muscle.

Immunohistochemistry for the different isoforms of slow and fast myosin has been used by several authors (e.g., Refs. [52,53,54,55]) to evaluate the fibre composition and perform fibre morphometry in skeletal muscle aging. Gannon et al. [52] combined immunofluorescence with two-dimensional gel electrophoresis, mass spectrometry and immunoblotting to demonstrate a drastic increase in the slow MLC-2 isoform and a decrease in fast MyHCs in the gastrocnemius muscle of 26-month-old rats, consistent with the idea of an aging-associated shift to a slower twitching fibre population.

Hester et al. [53] applied immunohistochemistry on vastus lateralis muscles from young (18–26 year-old) and old (66–78-year-old) healthy male humans, demonstrating that in old subjects an age-related remodelling occurs, with an increase in type I fibres and a decrease in the ratio of the type II to the type I fibre cross-sectional area, in the absence of age-related atrophy or changes in fibre composition.

By combining transcriptome profiling and immunofluorescence, Zhang et al. [54] compared, in 27-month-old mice vs. young 10-week-old mice, the fibre composition of the soleus and extensor digitorum longus as muscles with higher proportion of slow- or fast-twitch fibres, respectively. The authors found that fast/glycolytic fibres and slow/oxidative fibres responded differently to aging. In fact, in muscles mainly composed of slow fibres, the proportion of these fibres did not change, whereas a significant decrease in oxidative fibres occurred in muscles characterized by their lower content.

By combining Western immunoblotting and immunohistochemistry, Lin et al. [55] showed an increased age-related expression of cardiac α-MyHC isoform 6 [56] in the rectus femoris muscle of 24-month-old mice, with the greatest immunopositivity in degenerated myofibres.

The thick and thin filaments are arranged in the three-dimensional structure of the sarcomere, whose mechanical stability is ensured by an organized network of intermediate filaments, which interconnect adjacent myofibrils and link them to the sarcolemma and nucleus [57,58]. Desmin is the major intermediate filament in adult muscle, and it plays a key role in maintaining the spatial relationship between the contractile apparatus and other myofibre structural elements [59]. Ansved et al. [60] investigated the fast-twitch extensor digitorum longus and slow-twitch soleus muscles of young adult (5–6 months) and old (21–25 months) rats, demonstrating that immunolabelling for spectrin (a cell-membrane-attached cytoskeletal protein) did not change with respect to age in either muscle. Conversely, desmin immunostaining was heterogenous in the muscles from the old rats, the atrophic fibres showing an overall higher fluorescence signal and the normal-sized fibres showing heterogeneous staining. This suggested an age-associated increase in the inter-myofibrillar space close to the Z-line, where desmin is located.

### 2.2. Sarcolemma and Sarcoplasmic Reticulum

Myofibres are bounded by the sarcolemma. This plasma membrane acts as a mechano-sensor allowing the myofibre to interact with the connective tissue (the endomysium) surrounding each myofibre. The sarcolemma also allows the reception of the motor neuron stimuli at the endplate of the NMJ as well as its efficient propagation in the whole sarcoplasm through tubular invaginations called the transverse (T)-tubules. T-tubules form with the terminal cisternae of the sarcoplasmic reticulum the so-called triad, a specialized intracellular membrane structure where T-tubule depolarization is transmitted to the sarcoplasmic reticulum, thus inducing Ca^2+^ release. The sarcoplasmic reticulum is a complex system of cisternae and tubules surrounding myofibrils. It functions as reservoir regulating the intracellular concentration of Ca^2+^ and plays a crucial role in the excitation-contraction coupling [61,62]. Several proteins in the sarcolemma and sarcoplasmic reticulum are responsible for Ca^2+^ homeostasis. Briefly, dihydropyridine receptor α1 (DHPRα1), occurring in the T-tubule, changes its conformation following membrane depolarization and activates ryanodine receptor 1 (RyR1), located in the junctional face membrane (i.e., the portion of terminal cisternae facing the transverse tubules) and responsible for Ca^2+^ release in the sarcoplasm. Junctophilins (JPHs), located in the junctional face membrane, connect the sarcolemma to the sarcoplasmic reticulum and support the interaction between DHPRα1 and RyR1 [63]. The sarco-endoplasmic reticulum Ca^2+^ ATPase (SERCA), located in the sarcoplasmic membrane, is responsible for the Ca^2+^ re-uptake. The luminal Ca^2+^-binding proteins calsequestrin (CSQ) and sarcalumenin (SAR) act as a Ca^2+^-buffering complex and endogenous regulator of Ca^2+^ release. The Na^+^/Ca^2+^-exchanger (NCX, located in both the sarcoplasmic reticulum membranes and sarcolemma) and the plasmalemmal Ca^2+^-ATPase (PMCA) mediate the Ca^2+^ extrusion.

Since both the sarcolemma and sarcoplasmic reticulum play a central role in muscle cell contraction, they have been extensively studied to understand their contribution to the impaired functionality of aged muscles.

O’Connell et al. [64] investigated various sarcolemmal and sarcoplasmic reticular factors involved in Ca^2+^ homeostasis in the tibialis anterior, gastrocnemius and soleus muscles of young (3-month-old), adult (6-month-old) and senescent (30-month-old) rats. Based on immunofluorescence experiments, it was found that the myofibres expressing SERCA2 (mainly present in slow-twitch myofibres) increased in aged muscles. This is consistent with the increase in the proportion of slow-twitch muscle fibres, due to the preferential decrease in fast-twitch fibres in sarcopenia. Moreover, the RyR1 immunolabelling increased in aged fibres, whereas the positivity for DHPRα1, SAR, NCX and PMCA was reduced. These alterations may contribute to the impairment of ion homeostasis and triad signalling that occurs in sarcopenia [65,66].

Aging is accompanied by alterations in the sarcoplasmic reticulum, leading to the formation of tubular aggregates. As demonstrated by immunocytochemical studies on the gastrocnemius and extensor digitorum longus muscles of mice, these tubular aggregates only form in fast type IIB muscle fibres, increasing in number and size with age, and contain SERCA1 [67,68,69], CSQ [68,69], SAR and RyR1 [68]. Remarkably, the tubular aggregates occur in the skeletal muscle of old males only, their formation probably being under hormonal control. Recently, immunohistochemical studies demonstrated that both SERCA1 and SERCA2a, which were distributed in the sarcolemma of the gastrocnemius muscles of 3-month-old mice, ectopically accumulated in the tubular aggregates in 24-month-old mice [70]. Tubular aggregates were also strongly labelled for DHPRα1, RyR1, JPH1 and JPH2, which are normally located in the sarcoplasm and sub-sarcolemma. In addition, immunostaining for matrix metalloproteinase 2 was found to co-localize with JPH1 and JPH2 in the tubular aggregates, suggesting that proteolytic activity is taking place therein. On the other hand, no tubular aggregates or SERCA displacement were found in the aged soleus muscle of the same animals [70]. These findings demonstrate that, in the tubular aggregates, SERCA segregates together with various sarcoplasmic reticular factors responsible for Ca^2+^ homeostasis. However, it is unlikely that tubular aggregates are functionally active because SERCA is blocked in a non-functional configuration [69].

The skeletal muscle polypeptide JP-45, which locates in the junctional face membrane, was investigated in murine skeletal muscles for its distribution and interaction with other proteins involved in the excitation–contraction coupling. JP-45 was found to co-localize with RyR1 in the triad membranes, and JP-45 showed downregulation in aging [71]. Since JP-45 affects the functional expression of the voltage-dependent Ca^2+^ channel Cav1.1 and its downregulation decreases the maximum charge movement [72], it is likely that JP-45 depletion in aging contributes to the functional loss of skeletal muscle.

Na^+^-K^+^-ATPase (NKA) is a sarcolemmal enzyme responsible for the maintenance of Na^+^-K^+^ homeostasis and the modulation of contractile functions, and the phosphorylation of its α1-subunit regulates the enzyme activity. Zhang and Ng [73] investigated by immunofluorescence the age-related modifications of the α1-subunit phosphorylation in the rat gastrocnemius muscle, and they observed a decrease in the muscles of 30-month-old rats, according to previous reports on the functional alterations of NKA in advanced age [74].

Reis et al. [75] used immunofluorescence to investigate in the skeletal muscle the age-related modifications of the NKA accessory protein, phospholemman. Both NKA and phospholemman were present along the sarcolemma of rat gastrocnemius muscles, but the involvement of phospholemman in the age-related dysfunction of NKA was excluded, as no change in the immunolabelling was observed during aging.

The sarcolemma maintains its integrity by binding to the ECM through the sarcolemmal scaffolding, whose key elements are the dystrophin–glycoprotein complex and the integrin/focal adhesion complexes [76]. The sarcolemmal integrity in the soleus and extensor digitorum longus muscles of 6-, 30- and 36-month-old rats was investigated by Rice et al. [77] using immunofluorescence. In both muscles, dystrophin immunolabelling was localized in the sarcolemma, appearing uniform and continuous in young animals but discontinuous in old animals or even absent in very old animals. As expected, the immunoglobulin G was absent in young myofibres but was present after aging. α-dystroglycan was irregularly associated with the sarcolemma after aging, while β-dystroglycan showed a continuous sarcoplasmic signal in the young myofibres and a punctuate labelling pattern after aging. The immunoreactivity for α-sarcoglycan (a membrane-associated glycan) increased in the extensor digitorum longus muscle and decreased in the soleus muscle of the old rats. Taken together, these findings suggest that the integrity of the sarcolemma is diminished and its glycosylation altered with aging, with different effects in fast- and slow-twitch muscle types.

Ramaswamy et al. [78] studied, using immunohistochemistry, age-related changes in the rat extensor digitorum longus muscle. In the muscles of 30–38-month-old rats, dystrophin decreased and no upregulation of utrophin (a dystrophin homologue) occurred, while dysferlin (a membrane protein involved in vesicle trafficking and membrane repair) ectopically relocated from the sarcolemma to the sarcoplasmic reticulum, although maintaining the same positivity levels as in young muscles. These findings indicate that in old skeletal muscles, the ability to transmit force laterally is impaired, thus contributing to the loss of muscle strength in aging.

The presence and distribution of dystrophin, α-syntrophin (another dystrophin-glycoprotein complex element) and neuronal nitric oxide synthase (nNOS, which normally binds to syntrophin and is involved in muscle contraction and atrophy [79]) were studied by immunohistochemistry in the plantaris muscles of 6- and 24-month-old rats [80]. The results demonstrated that aging induces α-syntrophin and nNOS disassociation from the sarcolemma, although no quantitative change was noticed.

As a whole, the above reported results suggest that the compromised integrity of the sarcolemma and diminished mechano-signalling may play a role in the progress of sarcopenia.

Montagna et al. [81] performed an immunofluorescence study in mouse tibialis anterior and gastrocnemius muscles and in the human rectus abdominis muscle: nNOS and denitrosylase S-nitrosoglutathione reductase (GSNOR, an enzyme involved in regulation of NO flux) were found to be co-localized in the sarcolemma, and GSNOR showed a decrease in aged muscles, suggesting that aging is associated with a defective NO homeostasis that may contribute to muscle wasting.

Parkin is a protein involved in the ubiquitination process, which is essential for the proteasomal degradation of damaged or misfolded proteins. By an immunofluorescence analysis of human vastus lateralis muscles, Serdaroglu et al. [82] demonstrated that parkin is present in the sarcoplasm as well as in the sarcolemma, with age-related differences in the immunostaining intensity and pattern. In fact, in young (3.5–7-year-old) individuals, parkin was mostly located in the sarcoplasm, with a weak and punctate pattern, whereas in aged (45–65-year-old) subjects, the immunostaining was intense in the sarcoplasm and accumulated in the sarcolemma. Due to the protective function of parkin, it was hypothesized that the upregulation and redistribution of this protein in aged muscle may be required to face the cumulative effects of oxidative stress with increasing age.

Sarcolemma has also been studied with respect to age-related metabolic alterations. Kukreti and Amuthavalli investigated by immunofluorescence some insulin signalling molecules in the hindlimb muscles of 3- and 24-month-old mice [83]. The glucose transporter protein type-4 (GLUT4), which is responsible for the insulin-mediated uptake of glucose in skeletal muscle, was found to be located in the sarcolemma of both young and aged muscles, with a significantly reduced intensity in the muscles of the old mice. This indicates a perturbation in the insulin signalling pathway and may partially account for the loss of insulin sensitivity in the elderly [84].

### 2.3. Mitochondria

Mitochondria are the primary source of energy for cells, and in skeletal muscle fibres are organized in a highly dynamic three-dimensional reticulum that is functionally optimized to distribute energy and metabolites throughout the cell [85,86]. At least two different mitochondrial sub-populations are recognized in the myofibre, i.e., the sub-sarcolemmal mitochondria, which provide energy for active transport through the sarcolemma, and the inter-myofibrillar mitochondria, specialized in energy production for contractile activity [87]. The inter-myofibrillar mitochondria are strategically located in the narrow space between the myofibrils, in register with the Z-disk of the sarcomeres, and in relation with the Ca^2+^ release units formed by the T-tubule and two terminal cisternae of the sarcoplasmic reticulum [88]. This organization facilitates Ca^2+^ mitochondrial uptake and mitochondria-mediated regulation of metabolic activity [89,90].

Mitochondrial Ca^2+^ import is carried out by the mitochondrial calcium uniporter complex (MCU): this is a multimer, made of the MCU channel-forming protein and associated regulators [91,92], among which are the mitochondrial calcium uptake 1 (MICU1) and MICU3. When downregulated, MICU1 promotes muscle atrophy or weakness [93,94]. MICU3 has recently been detected in skeletal muscle [95] and is thought to enhance mitochondrial Ca^2+^ uptake by working with MICU1 [96]. MICU3 immunostaining was found to decrease in the gastrocnemius muscle of 26-month-old mice [97], accompanied by a reduced mitochondrial Ca^2+^ uptake. This suggests that the impaired mitochondrial Ca^2+^ handling induced by the downregulation of MICU3 could contribute to the dysfunction of aged skeletal muscle. In fact, MICU3 overexpression led to a recovery of the physical function and mass of skeletal muscles. In the gastrocnemius muscle, immunofluorescence experiments demonstrated a downregulation of sirtuin-1 (SIRT1), i.e., a NAD^+^-sensitive deacetylase associated with lifespan and believed to exert beneficial counteracting effects on aging [98]. MICU3 and SIRT1 expressions were positively correlated, thus suggesting that in the skeletal muscle of aged mice, impairment in mitochondrial Ca^2+^ homeostasis due to MICU3 downregulation may involve the antioxidant response partially mediated by the SIRT1 pathway.

Mitochondrial dysfunction, as typical hallmark of aging, can be caused by the overproduction of mitochondrial reactive oxygen species (ROS) [99], i.e., the by-products of the electron transport chain (ETC), which promote oxidative stress as a major contributor to skeletal muscle weakness and loss during sarcopenia [100,101,102]. The occurrence of mutations in mitochondrial DNA may affect mitochondrial respiration due to the defective expression of mitochondria-encoded protein subunits of the ETC complexes, thus disturbing oxidative phosphorylation, reducing ATP synthesis and increasing ROS generation [103]. Dysfunctional fibres in aged skeletal muscle may result from the accumulation of mitochondrial DNA deletions, resulting in ETC complex defects and a respiration deficit in fibre segments. Müller-Höcker et al. [104] performed a cytochemical and immunocytochemical study under light and electron microscopy on muscles from old monkeys and demonstrated age-associated defects of the subunits forming the ETC complex IV (cytochrome-c-oxidase) and complex III. This loss of enzyme proteins affected all the mitochondria in single fibres, as shown by ultrastructural immunohistochemistry.

By a microarray analysis, Herbst et al. [105] found an increased expression of many genes affecting metabolism, metabolic regulation and mitochondrial biogenesis in ETC abnormal muscle fibres of the vastus lateralis muscle from 33-month-old male rats. In addition, a focally increased immunohistochemical signal was demonstrated in ETC abnormal fibres for different protein products, i.e., the pro-apoptotic P53 upregulated a mediator of apoptosis, PUMA [106,107], DNA polymerase gamma (which is responsible for replication and repair of mitochondrial DNA) [108,109] and prohibitin (which works in the mitochondrial inner membrane as scaffold for proteins and lipids regulating mitochondrial metabolism) [110]. Using immunohistochemical analysis, Herbst et al. [105] also demonstrated, in ETC abnormal fibres, the presence of the activated form of adenosine monophosphate-activated protein kinase (AMPK), which mediates the cellular response to energetic stress and mitochondrial insults [111], and increases levels of peroxisome proliferator activated receptor α [112] and peroxisome proliferator-activated receptor gamma coactivator 1 α (PGC-1α [113]). This evidence suggests that a cellular response to the disruption of β-oxidation occurred in ETC abnormal fibres as a result of the loss of electron transport.

Using a multimodal immunohistochemical approach, Fu et al. [114] demonstrated that PGC-1α plays a role in regulating mitochondrial function in sarcopenia via the sestrin2-mediated messenger target of the rapamycin complex 1 pathway. Sestrin2 is a stress-inducible protein preventing ROS-associated pathologies [115] and is downregulated in human skeletal muscles with aging [116]. Using specific antibodies, Fu et al. [117] showed decreased levels of Sestrin2 and of the phospho-40S ribosomal S6 kinase 1 (S6K1, which contributes to mitochondrial dynamics, homeostasis and function) in the gastrocnemius muscle of 24-month-old wild-type and PGC-1α conditional knockout mice. The treatment with recombinant sestrin2 increased the phospho-S6K1 level in the two genotypes, thus supporting the role of PGC-1α in the sestrin2-mediated messenger target of the rapamycin complex 1 pathway regulation of mitochondrial function in sarcopenia.

### 2.4. Myonuclei

Myofibre is a syncytium containing hundreds of myonuclei that, evenly spaced, lie just below the sarcolemma [118,119]. Each myonucleus governs a portion of cytoplasm, thus optimizing transcriptional and translational activities and ensuring sustainable myofibre development and homeostasis. Each cytoplasmic portion under the control of a single nucleus is named a myonuclear domain [118,120,121,122]. Thus, the size and function of the myofibres strictly depend on the number and spatial distribution of the myonuclei [123], and several investigations have been performed to accurately estimate their number in aged skeletal muscles. In these studies, immunohistochemistry was widely used to precisely distinguish the myonuclei from the myogenic satellite cells, which are located close to the muscle fibres between the basement membrane and the sarcolemma.

Renault et al. [124] used an antibody against the cell–cell recognition glycoprotein neural cell adhesion molecule (N-CAM) to specifically detect satellite cells, distinguishing them from myonuclei in masseter and biceps muscle biopsies from young (20–28-year-old) and aged (58–83-year-old) humans. The combined use of antibodies against laminin (to detect the basal lamina) and N-CAM allowed Kadi et al. [125] to discriminate with a higher degree of accuracy the myonuclei in the human tibialis anterior muscle. Other research groups [126,127,128] applied immunofluorescence to quantify the myonuclei in human vastus lateralis muscles by labelling laminin and paired box domain 7 (Pax7, a transcription factor typical of satellite cells). Similarly, Brack et al. [129] quantified the myonuclei and the nuclei of satellite cells in myofibres isolated from mouse tibialis anterior muscles by performing fibre typing and labelling satellite cells for Pax7, MyoD and syndecan-4 (Syn4) (all transcription factors specifically expressed by satellite cells, see Section 3). Gallegly et al. [130] immunolabelled dystrophin to visualize the sarcolemma and identify the myonuclei in rat soleus muscles. Some of the above studies reported an increased number of myonuclei per fibre in aged subjects [125,126], suggesting that this might be due to a decrease in the myofibre volume, which is consistent with sarcopenia. Conversely, other studies described a decreased number of myonuclei [129], supposing a less efficient replacement of the myonuclei by satellite cell activation. Other studies did not find quantitative changes [127,128,130]. This inconsistency may be related to the different experimental models used (humans or animals, whose muscle features does not correspond exactly), to the subjects’ age (sarcopenia affects middle-aged, old and very old subjects differently) and to the muscle and/or fibre type considered. Moreover, erroneous counting of myonuclei may occur since in aged muscles they may form aggregates or chains [126,129]. In addition, the reliability of quantitative evaluations strictly depends on appropriate sample size.

The application of immunohistochemical techniques in the above studies also revealed changes in the myonuclear shape, which becomes more irregular with age [126,129], as well alterations in the size of the myonuclear domains [126], which can possibly impair the distribution of gene products and alter the turnover of contractile proteins [131].

Quantitative ultrastructural immunohistochemistry with gold-labelled antibodies was used on the biceps brachii and quadriceps femoris muscles from 28-month-old mice: decreased levels of RNA polymerase II and some nucleoplasmic factors responsible for transcription, splicing and processing of messenger RNA (mRNA) were demonstrated in the myonuclei, together with a nuclear accumulation/relocation of mRNA cleavage factors and polyadenylated RNA [132,133]. Similar results were obtained in 19-month-old Ts65Dn mice, a model of premature aging [134]. These findings suggest an impairment in the production and export to the cytoplasm of gene products in aged muscles. Interestingly, immunogold under transmission electron microscopy demonstrated that adapted aerobic exercise is able to re-activate myonuclear activity in the quadriceps and gastrocnemius muscles of aged mice [135].

The aberration of DNA methylation, i.e., the addition of a methyl group to the 5′ position of the cytosine pyrimidine ring, leading to the formation of 5-methyl cytosine (5-mC), is known to occur in aged skeletal muscle [136]. Quantitative evaluations of the immunolabelling for 5-mC allowed an evaluation of these age-related epigenetic changes in the myonuclei of the rectus femoris muscles from 28-month-old mice [137]. Lower nucleolar amounts of 5-mC were found in old compared to adult animals, suggestive of a loss of rRNA genes. In the same muscles, after immunolabelling, ribonuclease A (RNase A, which is responsible for the intranuclear degradation of RNA [138]) and actin (i.e., the motor protein regulator of RNA transcription and intranuclear motility [139]) were found to decrease, strongly indicating an impairment of RNA transcription and/or cytoplasmic export.

In a recent multidisciplinary study, Habiballa et al. [140] investigated the occurrence of senescent markers in the vastus lateralis muscle of men and women of increasing age (from 45 to 85 years), with the aim of exploring sex-specific associations between cellular senescence and physical function. Using an immunohistochemical approach, they found no relationship between age and some cellular senescence markers, namely, p16 (a regulating factor of the cell cycle), γH2A.X (a phosphorylated form of the histone variant H2A.X primary marker for DNA double-strand breaks) and lamin B1 (a protein of the nuclear envelope involved in chromatin stability and gene expression). High-mobility group box 1 (a protein involved in chromatin remodelling and transcriptional reprogramming during senescence) was the only senescence marker showing a tendency towards the loss in women, suggesting a sex-specific difference for this protein expression [140].

These results are consistent with a study on the age-related changes in the splice forms of *LMNA*, the gene encoding for the nuclear lamina protein lamin A/C. Progerin is a truncated protein isoform generated through inappropriate alternative splicing of *LMNA* transcripts. It is responsible for premature aging diseases [141] but is also present in normal tissues, where it is supposed to be implicated in the physiological aging process [142,143]. However, by using an anti-progerin antibody, Luo et al. [144] found only a weak immunofluorescence signal in the myonuclei of the human vastus lateralis muscle of subjects aged from 16 to 70 years, suggesting that progerin is not involved in the aging process of skeletal muscles.

Ruiz et al. [145] used immunofluorescence to investigate the impact of aging on a specialized population of myonuclei, the subsynaptic myonuclei, which are involved in the maintenance of the NMJ. To identify the subsynaptic myonuclei, three proteins were specifically detected, namely, nesprin 1 (which allows the distinguishing of the subsynaptic myonuclei from the Schwann cell nuclei), neurofilament 2H3/synaptic vesicle glycoprotein 2A (labelling nerve and presynaptic terminals) and the postsynaptic nicotinic acetylcholine receptors (stained with α-bungarotoxin). The authors quantified this specific myonuclear population in the gastrocnemius muscle of 6- and 28-month-old mice, demonstrating no age-related alteration in their level or distribution despite the degeneration of NMJ occurring in sarcopenia.

Table 1 summarizes the age-related alterations of the myofibre components described in this chapter. 

## 3. Satellite Cells

In skeletal muscle fibres, satellite cells lie between the basal lamina and the sarcolemma [146]. They are spindle-shaped myogenic postnatal stem cells characterized by a single heterochromatic nucleus and a very small amount of cytoplasm and number of organelles [147]. In adult muscles, satellite cells are usually quiescent but, upon physiological stimuli (e.g., exercise) or pathological stimuli (such as muscle denervation or mechanical injury), they are able to re-enter the cell cycle and proliferate, giving rise to myoblasts. In addition, in the so-called satellite cell niche, manifold physical or chemical factors (e.g., mechanical stress, growth factors, cytokines, oxidants and free radicals, altered ion concentration) can activate signalling cascades that target the satellite cell nucleus [148,149] and modify gene expression, finally leading to satellite cell activation. Some of the resulting myoblasts may revert to quiescence to preserve the satellite cell population (e.g., Refs. [150,151,152]), while others can repair existing myofibres or undergo cell fusion and generate new multinucleated syncytia, thus supporting the regeneration and plasticity of the skeletal muscle [153,154].

Due to their primary role in muscle growth, maintenance and regeneration, satellite cells have been widely investigated. In particular, age-related modifications in their number and/or myogenic potential have attracted the attention of researchers as possible contributing factors to sarcopenia.

In this view, the use of antibodies against myogenic transcription factors expressed in specific functional phases of satellite cells has allowed the immunohistochemical visualization of this cell population in skeletal muscles. Some examples are the already mentioned Pax7 (a marker of both quiescent and activated satellite cells), the myoblast determination protein 1 (MyoD, a marker of activated and proliferating satellite cells), the proliferating cell nuclear antigen (expressed concomitantly to MyoD), myogenin (a marker for satellite cell commitment), the myogenic factor 5 (Myf5, involved in the control of satellite cell differentiation) and syndecan 3 and 4 (Syn3 and 4, expressed in quiescent and early-activated satellite cells) [155,156,157,158,159]. Satellite cells can also be detected by their immunopositivity for other protein markers, i.e., the mitogen-activated protein kinases ERK1 and ERK2 (expressed in the cytoplasm of satellite cells, regardless of their ability to express specific myogenic regulatory factors [159]), the plasma membrane protein N-CAM (also known as CD56, which is expressed by satellite cells committed to differentiation [160]) or the transmembrane glycoprotein M-cadherin (which anchors satellite cells to the sarcolemma and is involved in the early phase of myoblast fusion into myotubes [161]).

Satellite cells were identified and quantified in various human or murine skeletal muscles by means of immunohistochemistry using anti-Pax7 antibodies [126,127,128,162], anti-MyoD antibodies [163] or anti-N-CAM antibodies [124,125,164,165], or by combining anti-Pax7 with anti-N-CAM antibodies [166], anti-Pax7 with anti-MyoD antibodies [167,168], anti-Pax7 with anti-MyoD and anti-Syn4 antibodies [129], or anti-Pax7 with anti-mitogen-activated protein kinases, anti-proliferating cell nuclear antigen and anti-myogenin antibodies [169]. The results of these studies were inconsistent as the number of satellite cells was found to decrease or to be unchanged with age, depending on the fibre type and species. This suggests that the number of satellite cells is not a primary contributing factor to sarcopenia, although the myogenic potential and nuclear activity of satellite cells seem to play a role in the reduced regeneration capability and in the resulting loss of muscle mass during aging. It should be also taken into consideration that satellite cell quantification may be affected by methodological limitations, as discussed for myonuclei (see Section 2.4).

The ultrastructural immunolabelling of phosphorylated RNA polymerase II, DNA/RNA hybrid molecules (that are specifically located at the transcription sites), heterogeneous nuclear ribonucleoproteins core protein, small nuclear RNP Sm-core protein and non-small nuclear RNP splicing factor SC35 (all splicing factors) and cleavage stimulation factors demonstrated that the transcription, splicing, cleavage and intranuclear transport of mRNAs decreases in the satellite cell nuclei of the biceps brachii and quadriceps femoris muscles of 28-month-old rats [165] as well as in the quadriceps femoris and gastrocnemius muscles of 28-month-old mice [135]. This evidence suggests that the responsiveness of satellite cells to activation and differentiation stimuli may be hampered in the elderly. Accordingly, Kurland et al. [170], by applying immunofluorescence with anti-Pax7 antibodies and the incorporation of modified thymidine analogue EdU^TM^ to label newly synthesized DNA, demonstrated a lower proliferation and differentiation capability in the satellite cells of the tibialis anterior muscles of 24–28-month-old vs. 4–8-month-old mice.

Fujimaki et al., by combining immunohistochemistry for Pax7, MyoD and Myf5 in murine gastrocnemius muscles, found a decrease in the satellite cells of muscles from 24-month-old mice, suggestive of a decline also in myogenic capability. However, after physical exercise, the Myf5 and MyoD were found to increase through wingless-related integration site/β-catenin signalling, demonstrating an activation effect on satellite cells [171]. Moreover, alterations in the mRNA pathways were partially restored, as demonstrated by immunogold studies [135]. Consistently, in a study carried out in the tibialis anterior muscles of 4–5-month-old and 25–27-month-old rats, the immunolabelling of M-cadherin, Ki-67 (a nuclear protein expressed in all phases of the cell cycle, except the quiescent G_0_ phase) and myogenin demonstrated that low-frequency electrical stimulation may induce a similar increment in the number of satellite cells in young and aged muscles, supporting the notion that muscle contraction has a stimulating effect to counteract the age-related decline in satellite cells’ functionality [172].

Autophagy, and especially mitophagy, plays a critical role in satellite cell senescence. This was demonstrated by García-Prat et al. [173] in a multidisciplinary study on mouse tibialis anterior muscle and human vastus lateralis muscle, using a panel of antibodies for the immunolocalization of Pax7, MyoD, myogenin, N-CAM, Ki-67, p16, γH2A.X and the autophagy markers microtubule-associated protein light chain 3 and lysosomal-associated membrane protein 1, together with histochemical staining for mitochondria and ROS. The results showed that quiescent satellite cells display a continuous autophagic activity, which declines during aging with a consequent accumulation of toxic waste and ROS that impairs cell functionality.

Lastly, in a recent study, Liu et al. [174] investigated, using immunofluorescence, the insulin receptor and the insulin upstream open reading frame isoform (INSU) in the human gastrocnemius muscle from 27- to 89-year-old subjects, demonstrating that these proteins are co-located with PAX7 and their expression increases in the muscles of old subjects. The authors hypothesized that the increase in insulin receptors and INSU may be a compensatory event for defective homeostasis and insulin resistance in aging. However, due to the possible interactions of INSU with the transcription factor Pax7, this occurrence could also inhibit satellite cell proliferation and regeneration.

Table 2 summarizes the age-related alterations of satellite cells described in this chapter.

## 4. Peri-Myofibre Components

### 4.1. Neuromuscular Junction

The NMJ is intended as the area of synaptic contact between a motor neuron branch and a specialized portion of the sarcolemma of its target muscle fibre, named the endplate. In detail, the motor neuron axon arborizes in branches, which distally enlarge, forming presynaptic boutons where vesicles filled with the neurotransmitter acetylcholine are present. The facing sarcolemma is rich in acetylcholine receptors. The NMJ synaptic organization can be altered in aging (e.g., Ref. [175]), and immunohistochemistry has often been used to assess the age-related deficits of NMJ architecture.

The antibody RT97, reacting with the non-myelinated constituents of presynaptic nerve terminals [176], was used by Deschenes et al. [177] in association with rhodamine-conjugated α-bungarotoxin to compare several pre- and postsynaptic NMJ features in the plantaris and soleus muscles of 21-month-old male rats vs. 10-month-old young adults. In detail, the following features were considered: the number of nerve terminal branches, the total length of those branches, the average length per branch, the branching complexity stained perimeter (total area including stained receptors and non-stained receptor regions), the cumulative areas occupied by acetylcholine receptor clusters and dispersion of the endplates. The results showed evident signals of age-related pre- and postsynaptic remodelling, which clearly indicate denervation. In their following studies [178,179,180], the same research group used immunocytochemistry to demonstrate that the pre-to-postsynaptic ratio remains constant in aging, and that resistance training causes muscle-specific pre- and postsynaptic remodelling. It was therefore suggested that NMJ components respond with unique levels of sensitivity to changes in activity.

AMPK is a regulator of autophagy and mitochondrial turnover implicated in NMJ dysfunction during aging [181,182] and is involved in NMJ maintenance and remodelling across the lifespan. With a multimodal approach, Ng et al. [183] investigated the role of AMPK in the glycolytic extensor digitorum longus and the slower, more oxidative soleus muscles of 3-, 10- and 22-month-old mice, and in age-matched knockout mice, null for skeletal muscle AMPK. The immunolabelling of neurofilament M and synaptic vesicle 2 was used to assess the presynaptic architecture, while the postsynaptic acetylcholine receptors were detected by fluorochrome-labelled α-bungarotoxin. It was found that several NMJ features (such as synapse fragmentation, ectopic formation of acetylcholine receptor clusters, axonal blebbing and sprouting axons) were significantly more numerous in the aged mice, and such an increase was even greater in knockout mice that exhibited an NMJ gene expression profile and phenotype resembling old animals. This suggests that AMPK has a critical role in the maintenance and plasticity of the NMJ.

As already recalled, Ruiz et al. [145] investigated the association between the number of subsynaptic myonuclei and the NMJ integrity: the subsynaptic myonuclei are located in the myofibre endplate, and are responsible for the local enrichment of the acetylcholine receptors and the other proteins required to maintain the structure and function of the NMJ (e.g., Ref. [184]). The authors demonstrated, using immunohistochemistry, that the number of subsynaptic myonuclei (identified by the immunopositivity for nesprin-1-α2 [185]) is not affected by the innervation status in the gastrocnemius muscle of 28-month-old aged mice compared to their young 6-month-old counterparts. Thus, the subsynaptic myonuclei, which provide support for the NMJ integrity, do not contribute to the NMJ deficits by varying in number with age, although the decreased NMJ integrity and the loss of innervation are known to contribute to age-associated muscle wasting [30,186].

### 4.2. Vascular Component

The skeletal muscle is highly vascularized. Its so-called microvascular unit is the pool of capillaries perfused by one terminal arteriole and drained by one venule. This unit participates in muscle homeostasis and supports muscle metabolism, being the smallest functional unit for blood flow regulation in skeletal muscles [187].

The immunohistochemical recognition of capillaries has long been performed by using antibodies recognizing the cluster of differentiation 31 (CD31), also known as platelet endothelial cell adhesion molecule-1 (e.g., Ref. [188]). In fact, this protein is highly expressed by endothelial cells and is an important constituent of the intercellular junctions in confluent vascular beds [189].

The CD31 immunolabelling of capillaries, accompanied by their morphological recognition, has allowed the definition of some indexes useful to study skeletal muscle aging [127,190], such as the capillary density (i.e., mean number of capillaries per square millimetre), capillary contacts (i.e., number of capillaries around a myofibre), capillary supply (i.e., myofibre area/capillary contact) and the capillary-to-fibre ratio.

Rivard et al. [191] investigated the hypothesis that angiogenesis may be affected by aging in two rodent models of hindlimb ischemia occurring at the age of 3 or 24 months. Using a combined immunohistochemical and biochemical approach, the authors demonstrated a lower expression of vascular endothelial growth factor (VEGF, an endothelial cell-specific mitogen) in the CD31-positive capillaries of old animals in comparison with their young counterparts, and determined that the cells responsible for VEGF expression include skeletal myocytes and CD3-positive T lymphocytes infiltrating ischemic tissues. This suggests that the strong age-associated reduction in migrating T cells in ischemic limbs would be a potential cause for the decreased expression of the angiogenic growth factor VEGF. The integrity of the endothelial cell function may therefore be compromised in aging skeletal muscle.

Coupling the terminal deoxynucleotidyl transferase fluorescein–deoxyuridine triphosphate nick end-labelling method for detecting apoptotic nuclei with CD31 immunohistochemistry, Wang et al. [192] studied the incidence of cell death in the gastrocnemius muscles of 2-, 11-, 22- or 25-month-old male mice. An age-related increase in cell death was found in the ECM stromal cells, which are located outside the myofibre basal lamina, and this increase was mostly due to the apoptosis of the capillary endothelial cells, without however an age-associated alteration in the capillary-to-myofibre ratio. As determined by semiquantitative immunohistochemistry, the increased apoptosis of the endothelial cells was concomitant with alterations in the levels of ECM angiogenic regulators, such as perlecan and a perlecan-domain V proteolytic product (see also Section 4.3).

In a recent study, Skoglund et al. [193] used an antibody recognizing the laminin subunit α-5 chain (laminin A) as a strong marker of capillaries compared with its weak myofibre labelling and evaluated the capillarization of the vastus lateralis muscle in lifelong-trained very old men, revealing that lifelong endurance training significantly improves capillarization.

### 4.3. Extracellular Matrix

The ECM is a highly dynamic network of collagens, glycosaminoglycans, proteoglycans, laminins, elastin and fibronectins [194], which structurally and functionally supports the muscle fibres, vessels and nerves. Embedding the myofibre, the ECM represents a key player in maintaining the muscle structure, and it constitutes a critical environment for the transfer of the muscle contraction force [195]. During the aging of skeletal muscle, alterations in the ECM deposition, architecture and composition take place [196,197], leading to increased muscle stiffness [197,198].

Immunohistochemistry has widely been used to specifically detect and quantify the ECM constituents of the skeletal muscle in aging subjects.

Ramaswamy et al. [78] demonstrated that interstitial ECM accumulates in the extensor digitorum longus muscle of 36–38-month-old rats, with an increased thickness of the myofibre basement membrane, where collagen type IV and laminin were immunolocalized.

Similar results were obtained by Lofaro et al. [199], who combined a proteomic approach with immunofluorescence labelling for collagen type VI, laminin and fibrillar type I collagen in the gastrocnemius muscle of adult (12-month-old) and aged (24-month-old) mice. The authors observed that in the old mice, the immunostaining intensity of the collagen type VI and laminin increased consistently with the proteomic data, while no quantitative difference was found for type I collagen, which however showed a less organized pattern than in the young mice.

In their already mentioned paper on the gastrocnemius muscle of 2- to 25-month-old mice, Wang et al. [192] investigated several ECM components, namely, collagen type VI and tenascin-X, which are located in the perimysium and endomysium, and the basement membrane collagen type IV, laminin and perlecan. The semi-quantitative immunohistochemical analyses revealed a maximum content of perlecan in 22-month-old mice followed by a decrease in the 25-month-old mice, with a concomitant increase in perlecan domain V (endorepellin) and LG1–LG2 peptides (produced by its proteolysis). Endorepellin is in fact subjected to limited proteolysis in presence of endothelial cells apoptosis, which was demonstrated here (as already reported in Section 4.2).

During aging, the regenerative capability of the skeletal muscle decreases, in part due to a reduction in the number and function of satellite cells, and changes in their ECM niche [200,201,202,203,204]. Schüler et al. [205] investigated the ECM composition in four muscles (gastrocnemius, soleus, tibialis anterior, extensor digitorum longus) with a different myofiber type composition in young (2–3-month-old), old (18–20-month-old) and geriatric (24–42-month-old) mice. Moreover, they studied the effect of aging on the communication between satellite cells and their niche using a deep proteomic analysis in association with immunofluorescence. An extensive remodelling of the ECM niche was observed during aging, with a reduction in the collagen type XIV α1 chain (Col14α1) and elastin, while asporin increased, and the secreted modular calcium-binding protein-2 (Smoc2) accumulated in the niche. Without excluding a contribution to the ECM remodelling by other cell types, such as immune cells, fibroblasts and satellite cells themselves, the authors pointed to fibro-adipogenic progenitors as the main source of the niche proteins affected during aging, as confirmed by the dual-immunofluorescence staining of Smoc2 and platelet-derived growth factor receptor α (the surface marker of the fibro-adipogenic progenitors).

Table 3 summarizes the age-related alterations of peri-myofibre components described in this chapter.

## 5. Conclusions

The advancement of technology in microscopy and image analysis, and the development of more and more specific antibodies and materials to process biological samples, have led to the wide application, under both light and electron microscopy, of immunohistochemistry in biology and medicine. This versatile technique is suitable for investigations at different resolutions, as it allows the detection of single molecular components at the tissue, cellular and sub-cellular level.

The wide range of immunohistochemical data reported in this review is proof of the primary role played by this long-established, yet modern, technique in the research on skeletal muscle aging. Immunohistochemistry, often in association with other analytical techniques, has allowed the identification of age-related molecular alterations to various myofibre components as well as in the peri-fibre environment. It was thus demonstrated that not only myofibres but also satellite cells, to the same extent as the connective, vascular and nervous components, are affected by aging, thus explaining the reduced regeneration capability of muscles in the elderly, and providing a mechanistic explanation of the loss of muscle mass and function in aged subjects.

Even about one century after the first, pioneering applications of labelled antibodies to detect molecular components in situ, immunohistochemistry still ranks high among the laboratory techniques in biomedicine.

The simultaneous detection of multiple markers on a single tissue section using a multiplex immunohistochemical approach may be envisaged as particularly promising to obtain a comprehensive view of the cell composition, function and interactions, with great potential for translational research and clinical practice [206]. In addition, three-dimensional histology and deep-penetrating immunohistochemistry [207] now make it possible to reconstruct microstructural details that can hardly be appreciated in two-dimensional images. As a consequence, a huge mass of structural and immunohistochemical features may be collected on a specific cell or tissue system, or on a pathological condition. To facilitate the management of such high-dimensional and complex data, helpful support may be given by machine-learning technologies that have been developed in recent decades and are rapidly gaining popularity as reliable automated diagnostic tools, especially for clinical applications [208]. During the learning process, information is extracted from images: this allows significant improvements in the classification tasks [209,210], helping pathologists to make increasingly accurate diagnoses.

With this progress in histochemical methods and technologies for image analysis and processing, immunohistochemistry will continue to be a unique, irreplaceable tool for basic and translational research, and for diagnostics.

## Figures and Tables

**Table 1:** Age-related alterations involving skeletal myofibre components as detected by immunohistochemical techniques.

Sarcomere	Sarcolemma and Sarcoplasmic Reticulum	Mitochondria	Myonuclei
Type II fibre atrophy	Increased SERCA2 and RyR1	Decreased MICU3, SIRT1, ETC complex III and IV, sestrin2 and S6K1	Altered number
Type I fibre enlargement	Decreased DHPRα1, SAR, NCX, PMCA, JP-45, phosphorylated Na^+^-K^+^-ATPase, dystrophin, GSNOR and GLUT4	Increased PUMA, polymerase γ, prohibitin, AMPK, PPARα and PGC-1α	Irregular shape
Muscle-specific reduced length of thin filaments	Accumulation of SERCA, DHPRα1, RyR1, JPH1 and JPH2 in tubular aggregates		Decreased nuclear domain size
Increased slow MLC-2, Myh6 and desmin	Altered distribution of α-dystroglycan, β-dystroglycan, α-sarcoglycan, dysferlin, α-syntrophin, nNOS and parkin		Decreased RNA polymerase II, splicing factors, RNase A, actin and nucleolar 5-mC
Decreased fast MyHC			Accumulation/relocation of mRNA cleavage factors and polyadenylated RNA

**Table 2:** Age-related alterations involving satellite cells as detected by immunohistochemical techniques.

Satellite Cells
Decreased or unchanged number
Decreased RNA polymerase II and splicing factors
Accumulation/relocation of mRNA cleavage factors
Decreased proliferation and differentiation capability
Decreased autophagic activity
Accumulation of ROS
Increased insulin receptors and INSU

**Table 3:** Age-related alterations involving peri-myofibre components as detected using immunohistochemical techniques.

Neuromuscular Junction	Vascular Component	Extracellular Matrix
Pre- and postsynaptic remodelling	Decreased capillarization	Increased thickness of basement membrane
Synapse fragmentation and ectopic formation	Decreased VEGF	Increased type VI collagen and laminin
Axonal blebbing and sprouting	Increased endothelial cells apoptosis	Altered distribution of type I collagen
		Altered expression and proteolysis of perlican
		Decreased Col14α1 and elastin in satellite cell niche
		Increased asporin and Smoc2 in satellite cell niche
